# An Update on Cutaneous Metastases of Internal Malignancies

**DOI:** 10.3390/medicina61091570

**Published:** 2025-08-31

**Authors:** Polixenia Georgeta Iorga, Andreea Dragomirescu, Lucian G. Scurtu, Olga Simionescu

**Affiliations:** 1 Faculty of Medicine, “Carol Davila” University of Medicine and Pharmacy, 020021 Bucharest, Romaniadana.simionescu@umfcd.ro (O.S.); 2 Department of Medical Oncology, Bucharest Emergency University Hospital, 011461 Bucharest, Romania; 3 Department of Dermatology I, Colentina Clinical Hospital, 020125 Bucharest, Romania

**Keywords:** skin metastases, cutaneous metastases, dermoscopy, biopsy, electrochemotherapy, palliative therapy, intralesional cryosurgery, breast cancer, lungs cancer, ovarian cancer

## Abstract

Skin metastases represent a rare finding in dermatological practice, but their presence signifies an advanced disease and usually portends a poor prognosis. They commonly arise as multiple painless nodules in patients with a cancer history. Differential diagnoses are challenging, and zosteriform metastases should not be mistaken for herpes zoster. Dermoscopy typically reveals a white, structureless pattern. A skin biopsy with routine hematoxylin–eosin staining is essential for an accurate diagnosis, while immunohistochemistry is particularly useful in cases of anaplastic tumors. Breast cancer is the most common cause of skin metastasis in women, and lung cancer is the most common in men. The life expectancy after diagnosis is generally low. Cutaneous metastasectomy, electrochemotherapy, and radiotherapy are generally regarded as beneficial for palliative purposes. Intralesional cryosurgery was found to be beneficial in a few case series. Systemic immunotherapy can induce the regression of cutaneous metastases in selected patients.

## 1. Introduction

Cutaneous metastases (CMs) are neoplastic infiltrations of the skin originating from distant malignant tumors. Their reported incidence ranges between 0.7% and 9%, accounting for only 2% of all skin cancers, and they occur in up to 10% of cancer patients. Usually, CMs appear as an evolution of the primary tumor, but they may also represent the initial sign of visceral cancers or announce cancer recurrence many years after the initial diagnosis. Certain tumors have a predilection for metastasizing to specific sites, which can guide the diagnostic approach for the underlying tumor. Moreover, the skin represents an easily accessible site for a biopsy specimen, with great implications in metastatic tumors of unknown origin [1,2,3,4,5]. The presence of CMs typically signifies advanced disease (stage III/IV) and carries a poor prognosis, with a median survival of 7.98 months; approximately 74% of patients present with concomitant visceral metastases [3].

CMs typically present as asymptomatic dermal nodules, although their clinical appearance can vary significantly. Disfigurement, impetiginization, fetor, ulceration, bleeding, necrosis, and drainage may worsen the natural evolution of skin metastases. It is essential to establish a prompt diagnosis to conduct an adequate treatment; hence, clinical recognition and skin biopsy of the suspicious lesions are crucial [5,6,7]. Dermoscopy is a rapid and non-invasive diagnostic tool for CMs, as demonstrated by Simionescu *et al*. In a 2024 study including 715 CMs, a characteristic structureless pattern was consistently observed, regardless of the primary tumor. Most CMs exhibited a single pattern, with pink and red coloration, irregular or dotted vessels, and occasional dots, globules, lines, streaks, lacunae, or milky-red structures [4]. CMs may develop through several pathophysiological mechanisms, including hematogenous spread, lymphatic dissemination, direct invasion, or accidental implantation during medical procedures. The metastatic process involves tumor cells detaching from the primary site, invading surrounding tissues, and entering the vascular or lymphatic circulation. Once disseminated, these cells must evade immune surveillance, extravasate at distant cutaneous sites, and establish secondary tumor growth within the skin [3,5,6].

Although CMs generally retain the histopathological features of the primary tumor, poorly differentiated or undifferentiated cells may also be present, in which case immunohistochemical studies are particularly valuable. Dermal pleiomorphic cells, mitotic figures, and neoplastic cells are characteristic findings of CMs. In contrast to primary cutaneous tumors, CMs do not originate from the epidermis and are completely separated from it [1,2,3,4,5,7,8]. This manuscript aims to summarize the main features of cutaneous metastases derived from internal cancers and to provide a comprehensive approach to their clinical and histological characteristics, differential diagnoses, and therapeutic options.

## 2. Materials and Methods

A comprehensive literature search was conducted in PubMed/MEDLINE, Scopus, and Web of Science up to August 2025, using the following terms and combinations: “cutaneous metastases,” “skin metastases”, “visceral cancer”, “breast cancer”, “lung cancer”, “mesothelioma”, “colon cancer”, “colorectal cancer”, “pancreatic cancer”, “stomach cancer”, “liver cancer”, “bladder cancer”, “prostate cancer”, “cervical cancer”, “ovarian cancer”, “endometrial cancer”, “cervical cancer”, “renal cell carcinoma”, “kidney cancer”, “”head and neck cancer”, “immunotherapy”, and “dermoscopy”. Boolean operators (AND, OR) were applied to refine searches. We included original articles, reviews, case series, and case reports published in English (and selected articles in Spanish and Portuguese with English abstracts) that described CMs of visceral malignancies, emphasizing clinical presentation, dermoscopic findings, histopathology, prognosis, and management. Exclusion criteria were studies on primary cutaneous malignancies (e.g., melanoma, cutaneous lymphomas, adnexal tumors), articles lacking sufficient clinical or pathological details, and non-peer-reviewed reports (abstracts without full text, editorial letters without cases).

After screening, a total of 181 references were selected, prioritizing landmark retrospective series, systematic and narrative reviews, and illustrative case reports of rare or unusual metastatic presentations. We extracted information on the primary tumor type and site, latency period, clinical morphology, dermoscopic and histopathologic features, metastatic sites, prognostic implications, survival data, and therapeutic approaches. This review is limited by the predominance of case reports and small series. The restriction to selected databases and mainly English-language publications may have introduced selection and language bias, while heterogeneity in reporting clinical and pathological findings limited direct comparisons across studies.

## 3. Cutaneous Metastases of Breast Cancer

**Figure 1:**
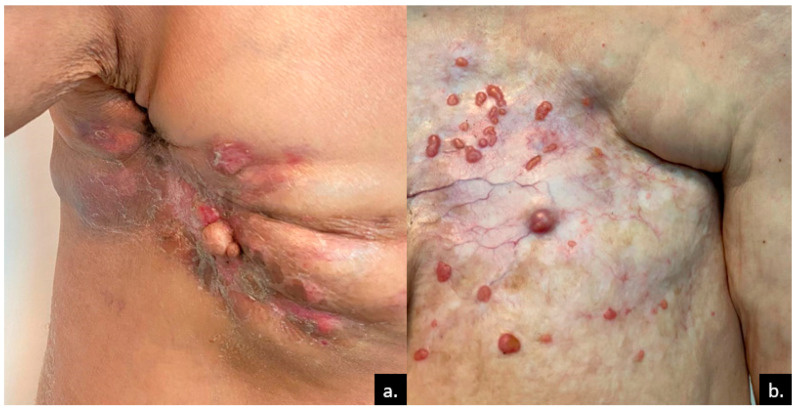
Skin metastases of breast cancer. (**a**) Metastatic nodules within the mastectomy scar of a 70-year-old female patient, accompanied by erythema, fissures, and crusts; (**b**) multiple papules and nodular skin metastases in a 60-year-old female patient who was undergoing chemotherapy for stage-IV breast cancer. The nodules are displayed on a fibrotic, telangiectatic plaque. Courtesy of Prof. Simionescu, from the personal clinical archive.

Breast cancer is reported to be the leading cause of CMs in women with non-melanoma solid tumors [5,6,7]. The most frequent form found to metastasize into the skin is ductal adenocarcinoma. CMs as a first sign of invasive ductal carcinoma are not uncommon. The most common site is the anterior chest wall, usually characterized by lymphatic spread to the overlying skin [6,7,8]. Other frequent sites are the scalp, neck, and abdomen. Although rare, CMs can be the initial evidence of breast malignancy. In exceptional cases, CMs may result from tumor cell seeding after a needle biopsy, particularly in subtypes with a papillary component. The most common clinical forms include multiple dermal nodules or papules. Typically, the nodules are painless, firm in consistency, and measure between 1 and 3 cm. The color usually resembles the skin, but can also be pink or red-brown [3,9,10,11,12,13,14,15,16,17] (Figure 1).

**Figure 2:**
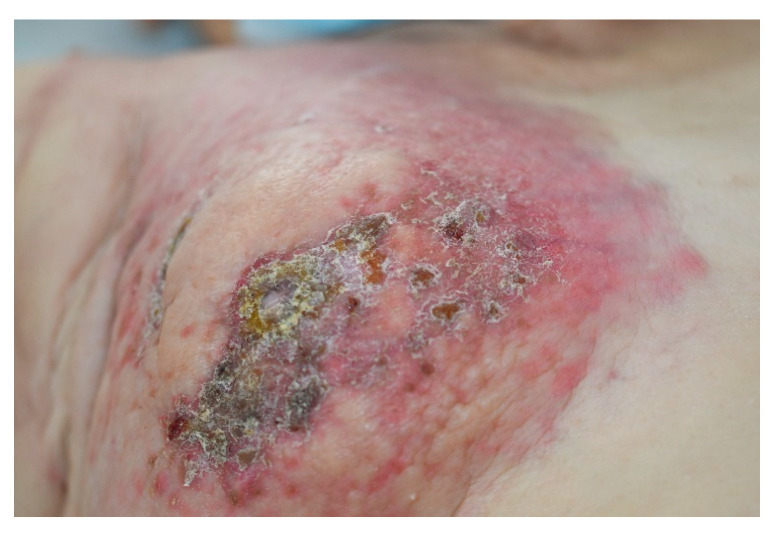
Dermatitis-like metastases, with few metastatic papules in stage-IV breast cancer. The metastatic large plaque presents with a secondary infection (impetiginization), with honey-like crusts. Courtesy of Prof. Simionescu, from the personal clinical archive.

**Figure 3:**
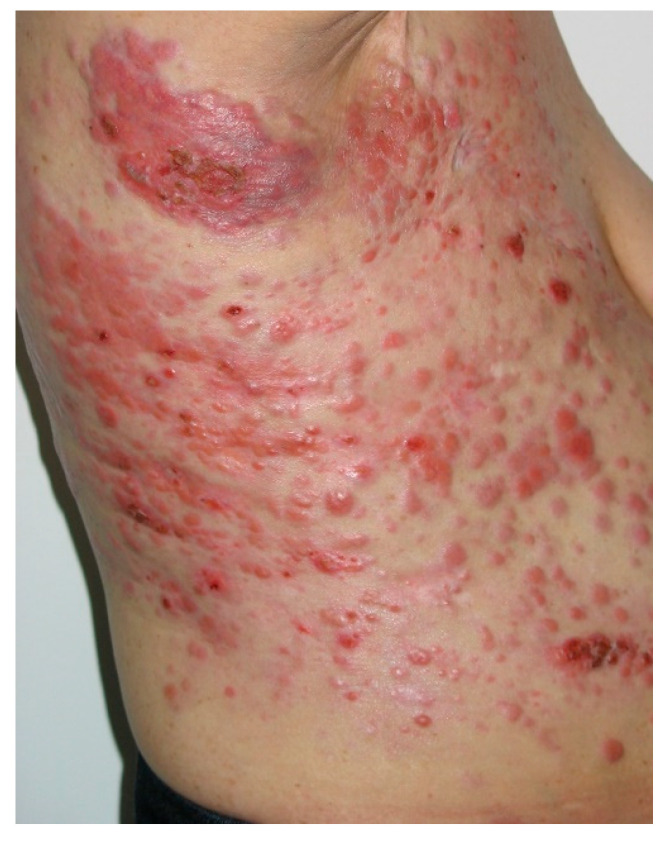
Hundreds of breast cancer metastases, involving the thorax and the abdomen, that tend to fuse into large plaques (incipient “en cuirasse”). Courtesy of Dr. Iorga, from the personal clinical archive.

Another relatively frequent breast cancer CM is *alopecia neoplastica* [14]. Other forms can include cellulitis, dermatitis-like metastases (Figure 2), “en cuirasse” (breastplate of armor) type (Figure 3), telangiectatic carcinoma, Paget-like metastases, or a zosteriform pattern [8,10,14].

Dermoscopy of breast cancer metastases predominantly displays a structureless pattern (98%), as well as a few blue-nevus-like and heterogeneous patterns. White (97.5%), blue (41.5%), and red (34.3%) are the most encountered colors. Irregular vessels account for the most frequent vascular subtype (13.6%), followed by comma (8.4%) and dotted vessels (7.65%). Dots and milky-red structures are a rare finding (2.47% and 1.23%, respectively) [4].

Microscopically, CMs are composed of neoplastic cells grouped in linear or glandular structures in a single layer between the collagen fibers, surrounded by fibrotic tissue. Immunohistochemistry provides positive tumor diagnostic markers such as cytokeratin (CK) 7, CK19, S100 protein, gamma-globulin, estrogen and progesterone receptors, and GCDFP-15 [12]. In most cancers, CMs appear in the advanced stage of tumor progression, and the prognosis is dependent on the characteristics of the primary tumor, such as the histological type and biological features [10].

Up to one-third of patients with metastatic breast cancer present with disease confined to the skin, a finding associated with a more favorable prognosis compared with visceral metastases. Loonkbill et al. reported an average survival of 31 months from the diagnosis of CMs to death. Overall, patients with breast-cancer-related CMs appear to have a better prognosis than those with CMs arising from other malignancies [3,7,9,11,15].

## 4. Skin Metastases of Lung and Pleural Cancers

### 4.1. Lung Cancer

Lung cancer is reported to be the leading cause of skin metastasis in men with non-melanoma solid tumors, with an incidence between 1.8% and 11.8% [5,7]. Usually, at the time of diagnosis, the skin is not the only metastatic region, and CMs often accompany other stage IV organ involvements. A higher rate of metastases to the skin due to adenocarcinoma and large-cell carcinoma is acknowledged. Small cell carcinoma has the lowest rate of cutaneous metastasis. The upper pulmonary lobes tend to have a higher rate of skin metastasis [18,19,20]. CMs may occur at distant sites such as the scalp, face, abdomen, and extremities, but they show a particular predilection for the supradiaphragmatic skin, most commonly involving the anterior chest and back overlying the primary tumor [7,18,19].

The clinical presentation of CMs includes several forms, most commonly the nodular subtype, but also inflammatory and scleroderma-like variants. Lesions typically measure 1–6 cm in diameter [19,21], and their color may range from flesh-toned, red, pink, or purple to bluish-black [21].

**Figure 4:**
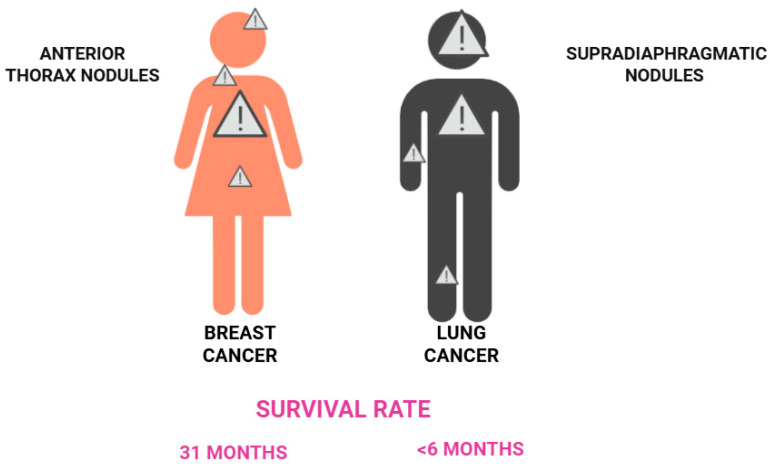
Leading causes of visceral CMs by gender (left, women; right, men) and their characteristics. Warning signs indicate the most frequent localizations of CMs. CMs: cutaneous metastases.

Less common forms include the ulcerated, vascular, plaque-like, papular, zosteriform, and erysipelas-like subtypes and, in some cases, scarring alopecia [21]. The neoplastic cells typically invade the lymphatic and vascular systems and are limited to the dermis and subcutaneous layer. The adenocarcinoma CMs are frequently moderately differentiated and may exhibit well-differentiated glandular structures or intracytoplasmic mucin [21]. Immunohistochemistry for lung adenocarcinoma shows positivity for CK7, TTF-1, Ber-EP4, and naspsin A [22,23] and rules out gastrointestinal, ovarian, and kidney cancer CMs [21]. CMs represent a poor prognosis marker, especially in patients with lung neoplasms, with a median survival of less than six months following diagnosis [7,19]. Figure 4 displays the main characteristics of CMs derived from breast and lung cancer.

### 4.2. Pleural Cancer (Mesothelioma)

**Figure 5:**
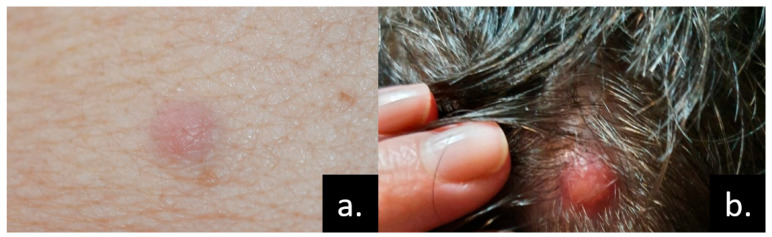
Skin metastases of pleuro-pulmonary cancers. (**a**) A pink, painless nodule localized on the upper abdomen of a 58-year-old patient with metastatic right lung adenocarcinoma; (**b**) painless metastatic nodule, with visible telangiectasia on the scalp of a 62-year-old female patient with mesothelioma. Courtesy of Prof. Simionescu and Dr. Iorga, from personal clinical archives.

Unlike lung cancer, malignant mesothelioma is a rare neoplasm and an uncommon cause of CMs. CMs most often occur near the primary tumor site. Periumbilical lesions, such as Sister Mary Joseph’s nodule, may represent metastatic spread from peritoneal malignant mesothelioma. Other reported sites include the chest, face, and scalp [24,25,26,27,28] (Figure 5). Unexpected metastatic sites can be encountered, such as the central nervous system or acral regions. The most common clinical presentations include multiple subcutaneous nodules, violaceous papules, and inflammatory plaques, often with progressive extension. The median interval from diagnosis of the primary tumor to the development of CMs is approximately six months. In some cases, however, CMs may represent the initial manifestation of the disease [28,29,30,31,32,33,34,35].

Hematoxylin and eosin staining typically displays the dermal proliferation of infiltrative epithelioid cells, pseudoglandular structures, cytologic atypia, and increased mitotic activity [24]. Immunohistochemistry shows positivity for calretinin (most specific for mesothelioma) and HBME-1 [29,33], CK5, CK6, orthokeratin, vimentin, and EMA (epithelioid membrane antigen) [22,34,35]. Electron microscopy can reveal uniform sinuous surface microvilli with desmosome junctions, tonofilaments, and intracellular lumina, with a length/diameter ratio of the microvilli usually greater than 11 [34].

## 5. Skin Metastases of Gastrointestinal Cancers

### 5.1. Colorectal Cancer

**Figure 6:**
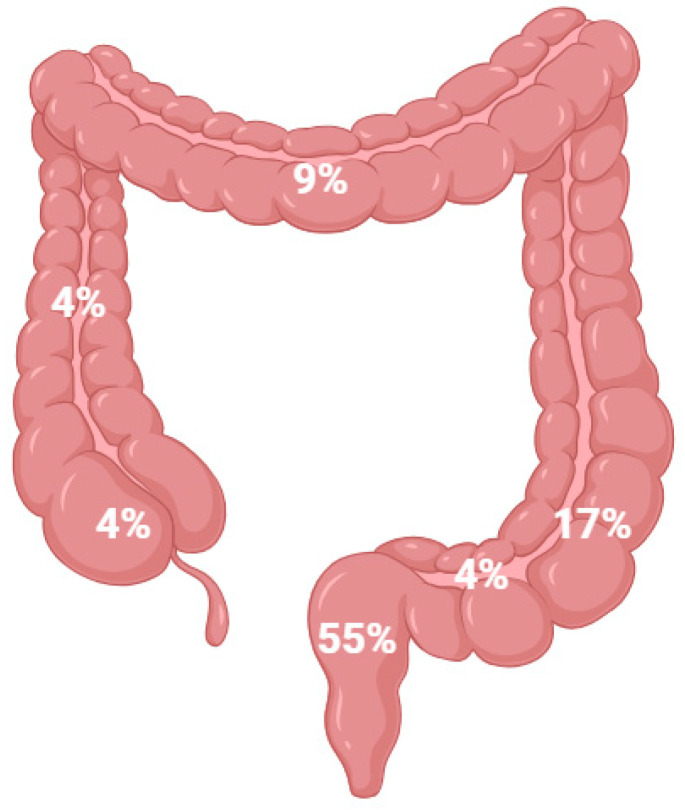
Distribution of CMs derived from colorectal cancer by topography. The left colon accounts for more than 75% of CMs.

Colorectal cancer is the second most common cause of skin metastasis for men with non-melanoma solid tumors (incidence of 11%). For women, the incidence is almost ten times lower (approximately 1.3%) and represents the sixth cause of cutaneous metastasis. The rectum is reported to be the first site of skin metastasis (55% of cases), followed by the sigmoid colon (17%), transverse colon (9%), rectosigmoid (7%), cecum (4%), and ascending colon (4%) (Figure 6) [36,37,38,39].

**Figure 7:**
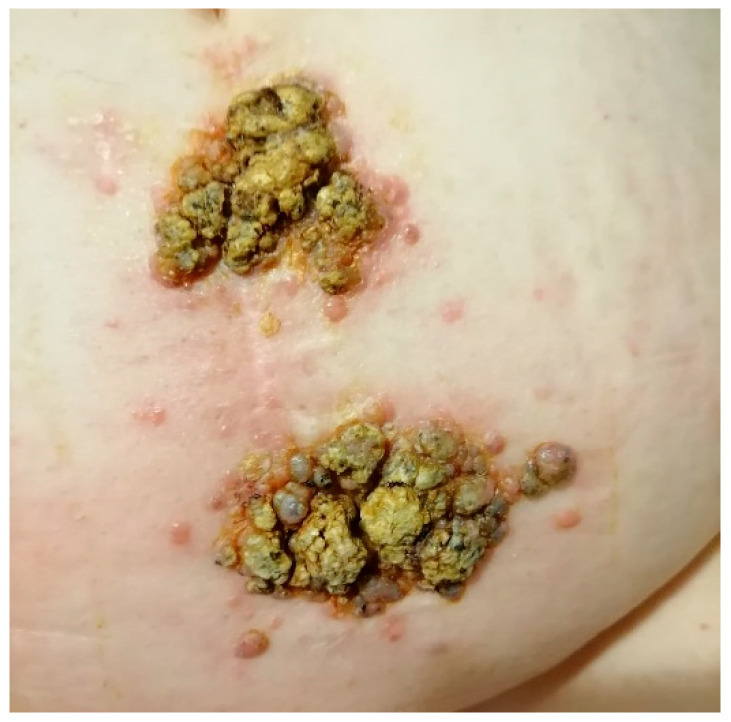
Incision scar for colon cancer, with metastatic papillomatous and verrucous tumors, covered with honey-like crusts, surrounded by satellite metastatic papules. Courtesy of Dr. Iorga, from the personal clinical archive.

CMs typically develop approximately two years after resection of the primary tumor and are usually associated with additional metastatic sites. The most frequent locations are the thoracic and abdominal regions, particularly along surgical incision scars (Figure 7). Less commonly, CMs may arise on the scalp or face [38,39,40].

Colorectal CMs commonly arise as painless, violaceous, or flesh-colored, firm nodules. They may occasionally be misdiagnosed and confounded with epidermal cysts, neurofibromas, lipomas, cicatricial morphea-like plaques, lymphomas, annular erythema, condylomas, and *elephantiasis nostra verrucosa*. The adenocarcinoma subtype is the most frequent source of these metastases, whereas mucinous adenocarcinoma and signet ring cell carcinoma are only rarely reported [5,7,12,36,37,38,39,40].

Colorectal CMs share certain histological features with the primary tumor. Most are well differentiated and mucin-secreting [37], although some may display more anaplastic characteristics, infiltrating the dermis and subcutaneous tissue without continuity with the epidermis [12]. Microscopically, they often exhibit glandular structures, with or without macronucleoli, but typically show goblet cells and intraglandular neutrophils [36]. Immunohistochemically, colorectal CMs are usually positive for CK20 and CDX2. Prognosis is generally poor, with most studies reporting a median survival of fewer than six months after diagnosis [11,36], although occasional reports have described survival of up to 18 months [7].

### 5.2. Stomach Cancer

Gastric cancer is a rare cause of CMs with an incidence below 1.5% and a higher risk reported for men; survival time is up to 16 months. The most frequent metastatic sites are the abdomen, neck, chest, and inguinal region. The most common histological subtype is adenocarcinoma. Skin metastases can manifest as solitary or multiple firm nodules, with colors ranging from red to violaceous. In most cases, stomach CMs are painless, having the starting point in the subcutaneous or dermal layer. Other clinical appearances can include erysipelas-like erythematous plaques, a cellulitis-like morphology, or a scleroderma-like type [7,41,42,43,44,45,46]. The proposed mechanisms of spread include hematogenous dissemination, lymphatic spread, direct invasion, and intraoperative implantation [46].

Poorly differentiated adenocarcinoma with signet-ring cell features is characteristic of stomach cancer CM [46]. Immunohistochemistry markers include CK20, carcinoembryonic antigen (CEA), EMA, CDX2, and HIK1083 [22].

### 5.3. Pancreatic Cancer

CMs from pancreatic cancer are rare, usually of the adenocarcinoma subtype, accounting for approximately2% of all metastases. The most frequent metastatic site is the umbilicus (known as the Sister Mary Joseph Nodule), although CMs can also occur at surgical scars, including incision or drainage sites [7,47,48,49,50,51,52,53,54]. The pancreatic regions most frequently associated with CMs are the head and uncinate process, followed by the pancreatic body [47,53]. Usually, tumors from the body or tail of the pancreas tend to metastasize to the umbilicus [50,53].

At diagnosis, CMs are often accompanied by other metastatic sites, although in some cases, they may precede identification of the primary tumor [52]. The most common clinical presentation is a solitary nodule, while less frequent forms include plaques, edema, or cutaneous thickening [47].

A histopathological examination typically reveals metastatic adenocarcinoma, usually poorly differentiated, infiltrating the dermis and subcutaneous tissue as invasive strands of pleomorphic cells with hyperchromatic, angulated nuclei. The immunohistochemistry examination is positive for diagnostic markers such as CK7, CK19, and carbohydrate antigen (CA)19-9, while the expression of CK20 is variable. Neither the sex nor the number of skin lesions impacts overall survival [47,48]. The median survival time from the diagnosis of CMs is reported to be five months [53].

### 5.4. Liver Cancer

Hepatocellular carcinoma (HCC) is a rare cause of CMs and accounts for less than 1% of all CMs. Cutaneous dissemination appears more frequently following a cirrhotic HHC. Metastatic carcinomatosis cirrhosis occurs when other organ malignancies infiltrate the liver and provoke fibrosis and should not be mistaken for HCC [7,54,55,56].

HCC skin metastases appear in the late stage of the disease, but they can rarely arise as a first clinical finding in an otherwise healthy patient. The most frequent sites are the head, trunk, and shoulders. The most common clinical presentation is asymptomatic, firm nodules with fast growth patterns and diameters less than 2.5 cm. HCC skin metastases can mimic pyogenic granuloma, granuloma teleangiectaticum, or a cutaneous abscess [56,57,58,59,60,61,62,63,64].

HCC is characterized by an affinity for Gallium, with arterial phase enhancement followed by a washout in the portal phase. It appears that skin metastases from HCC also have a higher uptake of gallium [59,61] when compared to normal tissues. Histopathological findings typically show abundant cellularity in a papillary or trabecular pattern, with neoplastic cells resembling hepatocytes but demonstrating a higher nucleus-to-cytoplasm ratio and prominent nucleoli [64,65]. Positive immunohistochemical staining for alpha-fetoprotein serves as a diagnostic marker [59]. CAM 5.2 is usually positive, whereas AE1 staining is negative [65,66]. CMs portend a poor prognosis, with a median 7-month survival rate [60,63].

## 6. Skin Metastases of Renal and Urogenital Cancers

### 6.1. Bladder Cancer

CMs from bladder cancer are rare, with an incidence below 4%. The direct implantation of the tumor cells into the granulation tissue of the surgical scar in the suprapubic area is a well-known cause of skin metastasis. The clinical presentation is usually nonspecific, most often manifesting as infiltrated plaques or nodules. These nodules are typically firm, round or oval, of variable size (median diameter of 1 cm), and mobile over the underlying structures [67,68,69,70,71,72,73,74,75,76]. There have also been reports of non-nodular forms, such as *carcinoma erysipelatoides* [68].

Urothelial cancer pathology, including infiltrating strands and nests of poorly differentiated carcinoma in the lower dermis, is encountered in more than 90% of CMs. Immunohistochemical studies reveal the positivity for the expression of CK7 and CK20 in 89% of urothelial bladder cancers [68,71]. The presence of cutaneous localization from urinary bladder cancer is highly correlated with large metastatic disease [74,75]. Survival after skin metastasis is low, with a maximum of 12 months [69]. Rare cases have reported longer survival, up to 34 months [70].

### 6.2. Prostate Cancer

Although prostate cancer has a high incidence in males, CMs are rare, with an overall incidence lower than 1%. The most frequent histological subtype is adenocarcinoma. Metastatic spread most commonly involves the inguinal region and abdomen, while less typical sites include the face, scalp [7,76,77,78,79,80,81], umbilical Sister Mary Joseph nodules [82,83], and trocar scars. A case report presented skin metastasis that mimicked extramammary Paget’s disease [84,85,86,87].

The clinical presentation consists of pink, dome-shaped, usually multiple nodules, with a firm consistency and a smooth surface. Other clinical findings are lymphedema or nonspecific, disguising rashes [78,88]. Histopathology reveals clusters of pleomorphic cells with glandular differentiation (adenocarcinoma) infiltrating the deep dermis. Immunohistochemistry should ideally include NKX3.1, PSA (prostate specific antigen), PAP (prostatic acid phosphatase), and neuroendocrine markers. NKX3.1, a prostate-specific homeobox gene product, is the most sensitive and specific marker for prostatic acinar adenocarcinoma, with a reported sensitivity of 98–100% and specificity exceeding 99% in metastatic lesions. This is particularly valuable in poorly differentiated tumors and metastatic sites, where PSA and PAP staining may be weak or absent, potentially leading to diagnostic ambiguity [79,83]. In some cases, the markers can be negative in the context of the massive lack of tumor differentiation [89,90]. The prognosis for patients with prostate cancer and CMs is reserved, with a median survival of seven months [78]. However, one case reported a higher survival status of 3.5 years [86]. It is hypothesized that the characteristics of the skin metastases, such as number, size, and location, are not responsible for the poor prognosis [88,91].

### 6.3. Ovarian Cancer

CMs of ovarian cancers have an incidence of approximately 3.5–3.8% and represent the fourth site of extra-abdominal metastasis in affected women. Their presence at the time of initial diagnosis is uncommon. The prognosis is poor, with a median survival of four to 12 months following diagnosis [91,92,93,94].

The most common affected area is the abdomen. Periumbilical Sister Joseph’s nodules are frequent, but other sites such as the pelvic region, thighs, or trunk can be involved. Orbital metastases have also been reported. Among histological subtypes, epithelial ovarian adenocarcinoma (especially serous papillary cystadenocarcinoma) most frequently metastasizes to the skin. Clinically, ovarian CMs usually present as multiple nodules less than 3 cm in diameter. While typically asymptomatic, some lesions may be painful or appear with herpetiform erythematous patterns, erythema annulare, or lymphangiosis carcinomatosa. Histopathology reveals neoplastic cells, organized in papillary groups, with hyperchromasia or enlarged nuclei, thinner nuclear membranes, enlarged nucleoli, and cytoplasmic vacuoles. Immunohistochemistry markers include progesterone and estrogen receptors, CK7, CA125, vimentin, and mesothelin, as well as Glut1 and PAX8 for a differential diagnosis [95,96,97,98,99,100,101,102,103,104,105,106].

### 6.4. Uterine Cancer

Uterine cancer is a rare source of CMs [7,107]. Adenocarcinoma is the most common subtype to metastasize [22,108,109], typically involving the lower abdomen, pelvic region, thighs, scalp, surgical scars, and the umbilicus. Additionally, less common sites have been described, such as paranasal sinuses or toes [109,110,111,112,113,114]. Clinically, the most frequent presentation is multiple subcutaneous nodules [108]. In addition, there have been reported patterns of zoster-like papular lesions [115]. Skin biopsy displays adenocarcinoma infiltration of the deep dermis and hypodermis. The immunohistochemistry exam can be positive for CK 7 and PAX8 [22]. Lookingbill et al. have reported a median survival of 34 months [7].

### 6.5. Cervical Cancer

Cutaneous metastases from cervical cancer are rare, with an incidence below 2%. Squamous cell carcinoma is the most common histological subtype to metastasize to the skin [107,116,117]. In some cases, the appearance of CMs can occur a few years after the diagnosis of the primary tumor. The abdomen, chest, and pelvic region represent the most common sites of CMs, followed by the extremities [107,118,119,120,121]. Rarely, metastatic sites can be the umbilicus [122,123,124] or the scalp [125,126]. The most common clinical presentation is multiple cutaneous nodules [118], while plaques, maculopapular forms, or inflammatory telangiectasia are less common. Skin biopsy reveals carcinoma (most frequent) and adenocarcinoma dermal infiltration. CK5, CK6, p63, and p16 are frequently positive in immunohistochemical studies [108,117,118,119,120]. The prognosis is poor, with a median survival of less than 9 months [116,117,118].

### 6.6. Renal Cancer

Between 4% and 8% of CMs originate from kidney cancer, most commonly from the renal clear cell carcinoma (RCC) [5,7,127,128,129,130]. In the majority of cases, CMs develop after the initial diagnosis of RCC; however, late recurrences may occur even ten years or more after nephrectomy. RCC-related CMs may also be detected at the time of diagnosis, though rarely beforehand. The most common metastatic site is the scalp, followed by the face and chest [7,131,132,133,134,135,136,137,138]. Less commonly, metastases may develop at the surgical nephrectomy scar [7].

**Figure 8:**
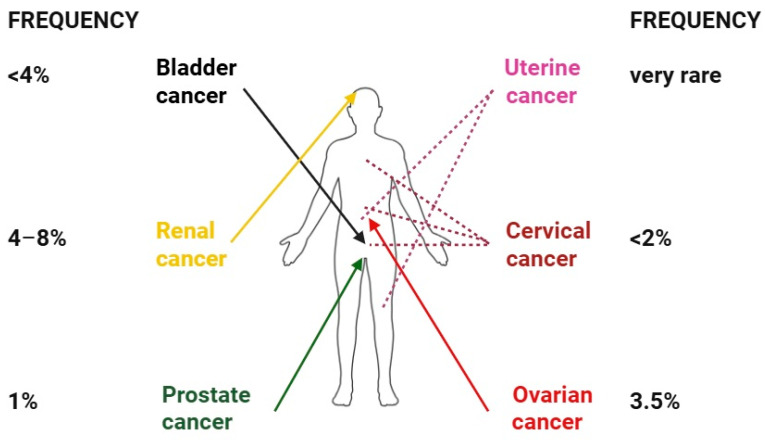
Frequency and topography of renal and urogenital CMs. While CMs from cervical and uterine cancers display a rather heterogeneous topography, the ovarian cancer CMs are confined to the periumbilical area, bladder and prostate cancers are confined to the pubic area/surgical scar, and renal cancer is confined to the scalp. CMs from renal cancer are most frequent among these organs. Multiple nodules are the most frequent clinical finding for all these CMs.

Clinically, RCC-related CMs usually present as multiple nodules, with an elastic or firm consistency, increased vascularity, and various colors (black, brown, or purple) [7,135]. Because of their vascular appearance, they may be misdiagnosed as cutaneous hemangiomas [131]. They are usually intra- or subcutaneous, oval- or round-shaped, with rapid growth. Histopathologically, tumor cells tend to be vacuolar, with clear cytoplasm, abundant in neocapillary formation, and with prominent lymphocytic infiltration. The layer above the secondary tumors is expected to be an atrophic epidermis [132,135]. Immunohistochemistry is a useful tool for differential diagnosis. The most frequent markers expressed in kidney tumors are EMA, vimentin, keratin, CD10, and CEA [135,136]. The median survival rate was reported to be between 7 and 21 months [7,133]. Figure 8 displays the topographical characteristics and frequency of CMs derived from urogenital and renal cancers.

## 7. Skin Metastases of Head and Neck Cancers

The incidence of CMs from head and neck squamous carcinomas appears to be below 2%. However, it represents 10% to 15% of all distant metastases. Contiguous involvement and tumor implantation after surgery are common. Squamous cell carcinoma is by far the most common histological subtype of head and neck cancers. CMs from head and neck malignancies are, however, exceedingly uncommon. Interestingly, among the limited cases reported, laryngeal atypical carcinoid (although rare as a primary tumor) appears disproportionately represented among those that metastasize to the skin. In suspected head and neck CMs, the immunohistochemical panel should include CK5/6 and EMA for squamous differentiation, p16 immunostaining as a surrogate for high-risk HPV infection (particularly in oropharyngeal primary tumors), and EGFR (epidermal growth factor receptor), which is commonly overexpressed in head and neck squamous cell carcinoma and can further support the diagnosis [16,139,140,141,142,143].

An important risk factor for developing skin metastases is the existence of a minimum of two cervical metastases or extracapsular extension in the cervical metastases. The existence of distant metastases without nodal involvement is a rare event [16]. Clinical presentations often involve asymptomatic forms, characterized by solitary or multiple subcutaneous or dermal nodules. The prognosis is very poor, as most patients die within three months after diagnosis [139].

## 8. Diagnostic Challenges and Differentials

The diagnosis of CMs can be particularly challenging, especially in patients without a known history of visceral malignancy. Among nodular presentations, 27% occur as solitary nodules and 23% as multiple nodules. Therefore, CMs should be considered in the differential diagnosis of indolent skin nodules, and biopsy is warranted when clinical suspicion is high [144,145]. A study found that 71% of CMs are correctly assigned during preoperative diagnosis, while the others were mistaken for common benign tumors (epidermal cyst, hemangioma, pyogenic granuloma, and zoster) [3].

Zosteriform metastases (ZMs) are CMs that occur in a dermatomal distribution. The pathophysiological mechanism of ZMs remains unclear and includes lymphatic and neural spread, a Koebner phenomenon at the site of a previous zoster infection, and the surgical implantation of tumor cells [146]. A meta-analysis found the following cancer distributions for ZMs: melanoma (18%), lymphoma (14%), breast cancer (12%), squamous cell carcinoma (12%), digestive cancers (10.7%), respiratory cancers (10.7%), urinary tumors (7%), and other cancers (14%). Hence, visceral cancers altogether are responsible for most ZMs (more than 40%) [147]. CMs can clinically mimic skin infections, inflammatory disorders, and other skin tumors (Table 1) [1,2,3,5,6,7,148,149,150].

**Figure 9:**
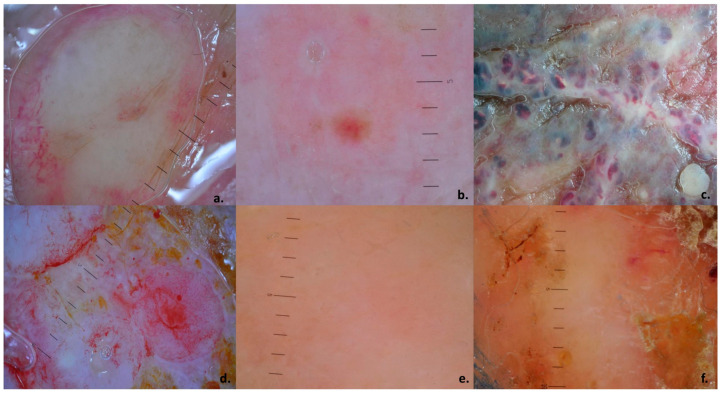
Dermoscopy of various skin metastases: (**a**) breast cancer, structureless white nodule with polymorphous and arborescent vessels in the periphery; (**b**) breast cancer, dotted and comma vessels and a central milky-red structure; (**c**) breast cancer, a large metastatic plaque containing multiple nonhomogeneous CMs (colors-white, blue, red, pink, purple); (**d**) oropharyngeal squamous carcinoma, a large plaque containing multiple metastases with structureless patterns, various colors (predominant white, red, pink, tan, yellow); (**e**) ovarian cancer, skin colored, structureless lesion with dotted vessels on a subtle erythematous background; (**f**) ovarian cancer, structureless, tan pattern with arborescent vessels and a peripheral yellow scale. Dermoscopy images were acquired with a Heine Delta 20+ dermatoscope (Heine Optotechnik, Herrsching, Germany) coupled with a Nikon D90 via Heine SLR adapter; scale bar: 1 mm. From the digital dermoscopy collection of Prof. Simionescu.

**Figure 10:**
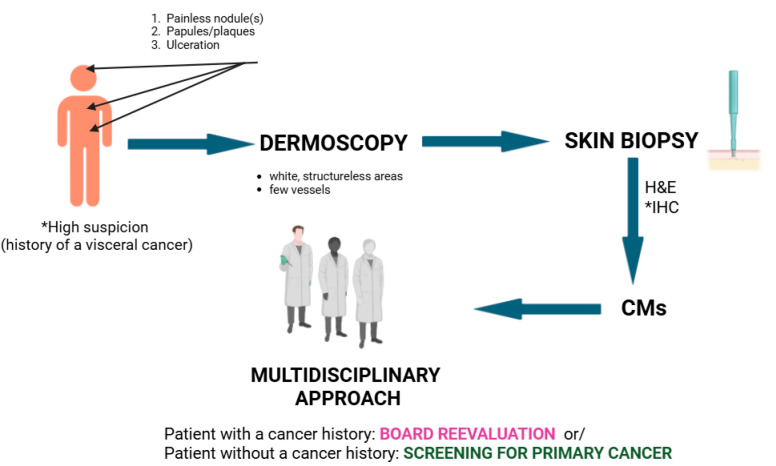
Diagnostic management of patients with CMs. The suspicion is high (*) in patients with a known history of visceral cancer. Skin biopsy is mandatory, preferably under dermoscope guidance. If the patient was known for a visceral cancer, he will undergo multidisciplinary board reevaluation and staging. If the patient does not have a cancer history, screening for primary cancer, including imaging and serology, will be conducted. H&E: hematoxylin and eosin; IHC: immunohistochemistry.

A CM suspicion should be raised in patients with a prior history of internal malignancy who develop firm, painless nodules (or papules/plaques/ulceration), commonly on the scalp, chest, and abdomen. The differentials should be excluded (clinical examination, dermoscopy); a skin biopsy is mandatory, and the biopsy site should ideally be selected under dermoscopic guidance (Figure 9). Once the diagnosis of CMs is confirmed, patient management should be discussed within a multidisciplinary team, including oncology, dermatology, and pathology specialists (Figure 10).

Table 2 summarizes the main clinical and immunohistochemical features of the CMs derived from internal malignancies.

## 9. Current Treatment Advances

Conventional surgery (metastasectomy) is commonly recommended for solitary skin metastases, with the aim of improving quality of life and regional functionality and reducing morbidity. Other established treatment modalities include electrochemotherapy, radiotherapy, intralesional and topical chemotherapy, and various combined approaches [151].

The electrochemotherapy technique combines the administration of a chemotherapy agent (usually bleomycin) with the local delivery of electric impulses (electroporation). The electric discharge increases the local permeability of the cellular membranes, with promising results regarding loco-regional disease control, especially in breast cancer metastases. In one study, bleomycin-based electrochemotherapy achieved a complete response rate of 46% in a palliative setting, irrespective of the tumor type. A novel variation of this technique combines electroporation with the local administration of CaCl_2_ (Ca-EP), which has shown promising results. Ca-EP was not found to be inferior to bleomycin and may be indicated in the future for patients who cannot receive chemotherapeutic molecules due to safety concerns [152,153,154].

Radiotherapy is primarily used for palliation, aiming to reduce local symptoms such as pain, weeping, fetor, and bleeding, rather than to decrease the size of large cutaneous or subcutaneous tumors. Treatment is typically brief and delivered in high individual doses of radiation, with palliative intent. Radiotherapy provides effective analgesia at low doses, with symptom relief reported even after a single 8 Gy fraction. Ulcerations usually heal within weeks [155,156].

Intralesional cryosurgery (ILC) is a novel therapeutic approach to CMs. The technique involves inserting a cryoprobe into the center of the tumor mass, followed by the passage of a cryogen through the probe until the lesion is visibly frozen, forming a surrounding frozen halo. ILC has shown benefits for CMs originating from breast, prostate, hepatic, non-small cell lung, and esophageal cancers [157,158,159,160].

## 10. Novel Systemic Therapies

Since the appearance of CMs usually reflects advanced disease, it often coincides with the availability of biomarker-driven systemic treatments that may prolong survival and improve quality of life. The choice of therapy depends on the molecular profile of the primary tumor.

In non-small cell lung cancer (NSCLC), molecular testing for EGFR mutations, ALK or ROS1 rearrangements, BRAF V600E mutations, and KRAS G12C mutations is now standard practice. Patients with EGFR-mutant NSCLC benefit from tyrosine kinase inhibitors such as osimertinib [161]. High PD-L1 expression identifies candidates for immune checkpoint inhibitors such as pembrolizumab or cemiplimab, which improve overall survival in advanced disease [162].

HER2-positive breast tumors are treated with targeted agents including trastuzumab, pertuzumab, and trastuzumab deruxtecan, which significantly improve progression-free and overall survival [163]. Hormone-receptor-positive tumors respond to endocrine therapy combined with CDK4/6 inhibitors (palbociclib, ribociclib, abemaciclib) [164]. In triple-negative breast cancer, PD-L1 positivity predicts the response to checkpoint inhibitors, such as atezolizumab or pembrolizumab [165].

Androgen deprivation therapy (ADT) remains the mainstay for metastatic prostate cancer, with second-generation androgen receptor antagonists (enzalutamide, apalutamide) and androgen synthesis inhibitors (abiraterone) improving outcomes [166]. Tumors harboring DNA repair gene alterations (BRCA1, BRCA2, ATM) are candidates for PARP inhibitors such as olaparib or rucaparib [167]. Radioligand therapy with lutetium-177–PSMA-617 has shown benefits for PSMA-positive metastatic castration-resistant prostate cancer [168].

In metastatic colorectal cancer, anti-EGFR monoclonal antibodies (cetuximab, panitumumab) are indicated for RAS wild-type tumors, while HER2 amplification identifies another actionable subgroup [169]. BRAF mutations can be addressed through targeted therapy with tirozinkinase inhibitors (encorafenib) and cetuximab [170]. Microsatellite instability-high (MSI-H) or mismatch-repair-deficient (dMMR) colorectal and gastric cancers respond well to PD-1 inhibitors [171]. Trastuzumab is standard for metastatic HER2-positive gastric or gastroesophageal junction adenocarcinomas [172].

In ovarian cancer, PARP inhibitors (olaparib, niraparib) are effective in BRCA-mutated or homologous-recombination-deficient metastatic disease [173]. In cervical cancer, pembrolizumab is approved for PD-L1-positive recurrent or metastatic tumors [174]. The addition of bevacizumab to chemotherapy has improved outcomes in both cervical and ovarian cancers [175].

Most head and neck squamous cell carcinomas overexpress EGFR, and cetuximab remains the primary targeted agent used in combination with radiotherapy or chemotherapy [176]. PD-1 inhibitors (nivolumab, pembrolizumab) are approved for recurrent or metastatic disease, especially in PD-L1-expressing tumors [177].

Although CMs usually reflect advanced disease, selected patients may experience the regression of these lesions under immunotherapy [178,179]. This observation emphasizes the relevance of molecular profiling and individualized therapy in optimizing outcomes.

Nonetheless, the efficacy of novel systemic therapies is often limited in patients with cancer cachexia, a syndrome affecting up to 80% of those with metastatic disease. Cachexia reflects profound tumor-driven alterations in neuroendocrine and immune homeostasis. Tumors may release hypothalamic and pituitary hormones, POMC-derived peptides, glucocorticoids, melatonin, leptin, acetylcholine, biogenic amines, and pro-inflammatory cytokines, which act both locally and on central regulatory axes such as the hypothalamic–pituitary–adrenal system. These mediators reset the energy balance, promote catabolism, and drive muscle wasting, while systemic inflammation further disrupts drug distribution and metabolism. As a result, cachexia not only accelerates clinical decline but also reduces tolerance and responsiveness to targeted agents and immunotherapy [180,181].

## 11. Conclusions

CMs of visceral cancers have an overall low incidence among skin cancers, but they display a poor prognosis and low survival rates. Still, patients with CMs of breast and uterine cancer survive up to two to three years after diagnosis. CMs most commonly arise as nodules, and other primary skin lesions such as papules, macules, plaques, and ulcerations are encountered.

Clinical recognition is essential, but is challenging, especially in patients without a history of visceral neoplasia. Zosteriform cutaneous metastases may be mistaken for zoster, and numerous differential diagnoses should be considered. Their early diagnosis can serve as a tool in staging the original cancer and reducing overall morbidity. Dermoscopy of CMs generally reveals a white, structureless pattern with few, irregular vessels. Still, histopathology and immunohistochemistry are the gold standard for a definitive diagnosis.

Surgery, classical bleomycin electrochemotherapy, and the novel calcium electrochemotherapy are regarded as generally safe and effective for reducing the disease burden and improving the quality of life for these patients. Radiotherapy is an analgesic and promotes healing of the ulcerated metastatic plaques. Intralesional cryosurgery was recently found to be beneficial in CMs. Immunotherapy may achieve the regression of CMs in selected patients, offering improvements in survival and quality of life.

## Figures and Tables

**Table 1:** Differential diagnosis of CMs.

Category	Differentials
**Inflammatory**	- Reactive granulomatous dermatitis - Sarcoidosis - Rheumatoid nodules - Erythema nodosum - Panniculitis - Pseudolymphoma - *Lupus erythematosus profundus* -Vasculitis - Morphea (nodular subtype) - Lichen nitidus
**Infectious**	- Bacterial abscesses - Tuberculosis cutis (scrofuloderma, *lupus vulgaris*) - Fungal infections (blastomycosis, histoplasmosis, coccidioidomycosis) - Leishmaniasis - Nocardiosis - Bacterial folliculitis - Bullous impetigo - Actinomycosis - Herpes zoster (for ZMs)
**Benign tumors**	- Lipoma - Dermatofibroma - Sebaceous cysts - Syringoma - Hemangioma - Fibroma - Seborrheic keratosis - Pyogenic granuloma - Glomus tumor
**Malignant tumors**	- Cutaneous lymphomas (mostly B cell type) - Basal cell carcinoma (nodular subtype) - Squamous cell carcinoma - Melanoma - Merkel cell carcinoma - Angiosarcoma

**Table 2:** Frequent clinical appearance and immunohistochemistry features of skin metastases of various visceral malignancies. EMA: epithelioid membrane antigen; CEA: carcinoembryonic antigen; PSA: prostate-specific antigen; PAP: prostatic acid phosphatase; EGFR: epidermal growth factor receptor.

Visceral Carcinoma	Localization	Clinical Appearance	Immunohistochemistry Markers	Life Expectancy After Diagnosis (Months)
Breast [7,8,9,10,11,12,14]	Anterior chest wall	Nodules	CK7, CK19, S100, CAM 5.2, gamma-globulin, estrogen receptor, progesterone receptor, GCDFP-15	31
Lung [19,21,22,23]	Not specific	Not specific	CK7, CAM 5.2, TTF-1, Ber-EP4, naspsin A	<6
Mesothelioma [24,25,26,27,28,34,35,36]	Abdomen	Sister Mary Joseph Nodules	Calretinin, HBME-1, CK5, CK6, orthokeratin, vimentin, EMA	N/A
Colorectal [7,12,36,37,38,39,40]	Abdominal surgery scar	Nodules	CK19, CK20, CDX2, CK20, CAM 5.2, CEA, Ber-EP4	<6
Stomach [7,22,41,42,43,44,45,46]	Not specific	Nodules	CK20, CEA, EMA, CDX2, HIK1083, Ber-EP4, CAM 5.2	<16
Pancreas [7,47,48,49,50,51,52,53]	Umbilicus	Sister Mary Joseph Nodules	CK7, CK19, CA 19-9, Ber-EP4, CAM 5.2	5
Liver [7,56,57,58,59,60,61,62,63,64]	Not specific	Nodules	alfa-fetoprotein, CAM 5.2	7
Kidney [7,131,132,133,134,135,136,137,138]	Scalp	Nodules	CK7, EMA, vimentin, keratin, CD10, CEA	<21
Bladder [67,68,69,70,71,75,76]	Not specific	Not specific	CK7, CK20	<12
Prostate [7,76,77,78,79,80,81,83,86,89]	Inguinal region, abdomen	Nodules	NKX3.1, PSA, PAP, neuroendocrine markers, Ber-EP4, CAM 5.2	7
Ovaries [91,92,93,94,100,101,102,103,104,105,106]	Abdomen	Nodules	progesterone receptors, estrogen receptors, CK7, CA125, vimentin, mesothelin, Glut1, PAX8	<12
Uterus [7,22,109,110,111,112,113,114]	Not specific	Nodules	CK7, PAX8	34
Cervix [108,116,117,118,119,120,121]	Not specific	Nodules	CK5, CK6, p63, p16	<9
Head and neck [16,139,140,141,142,143]	Neck, face	Nodules	CK5, CK6, p16, EMA, EGFR	3

## References

[1] Fernández-Antón Martínez M.C., Parra-Blanco V., Avilés Izquierdo J.A., Suárez Fernández R.M. (2013). Cutaneous metastases of internal tumors. Actas Dermosifiliogr..

[2] Sleeman J.P., Nazarenko I., Thiele W. (2011). Do all roads lead to Rome? Routes to metastasis development. Int. J. Cancer.

[3] Hu S.C., Chen G.S., Lu Y.W., Wu C.S., Lan C.C. (2008). Cutaneous metastases from different internal malignancies: A clinical and prognostic appraisal. J. Eur. Acad. Dermatol. Venereol..

[4] Simionescu O., Petrică M., Avram A.M., Costache M., Scurtu L.G., Tudorache S.I., Iorga P.G., Grigore M. (2024). Dermoscopy of skin metastases in advanced cancer-systemic (visceral, hematologic) and cutaneous. Front. Med..

[5] Strickley J.D., Jenson A.B., Jung J.Y. (2019). Cutaneous metastasis. Hematol. Oncol. Clin. N. Am..

[6] Gan E.Y., Chio M.T., Tan W.P. (2015). A retrospective review of cutaneous metastases at the National Skin Centre Singapore. Australas. J. Dermatol..

[7] Lookingbill D.P., Spangler N., Helm K.F. (1993). Cutaneous metastases in patients with metastatic carcinoma: A retrospective study of 4020 patients. J. Am. Acad. Dermatol..

[8] Wong C.Y., Helm M.A., Kalb R.E., Helm T.N., Zeitouni N.C. (2013). The presentation, pathology, and current management strategies of cutaneous metastasis. N. Am. J. Med. Sci..

[9] Sleeman J.P. (2000). The lymph node as a bridgehead in the metastatic dissemination of tumors. Recent. Results Cancer Res..

[10] Patra S., Khandpur S., Khanna N., Jain D. (2018). Angioma like carcinoma telangiectoides: An unusual presentation of breast carcinoma metastasis. Indian. J. Dermatol. Venereol. Leprol..

[11] Schoenlaub P., Sarraux A., Grosshans E., Heid E., Cribier B. (2001). Survival after cutaneous metastasis: A study of 200 cases. Ann. Dermatol. Venereol..

[12] Bittencourt Mde J., Carvalho A.H., Nascimento B.A., Freitas L.K., Parijós A.M. (2015). Cutaneous metastasis of a breast cancer diagnosed 13 years before. An. Bras. Dermatol..

[13] Araújo E., Barbosa M., Costa R., Sousa B., Costa V. (2020). A first sign not to be missed: Cutaneous metastasis from breast cancer. Eur. J. Case Rep. Intern. Med..

[14] De Giorgi V., Grazzini M., Alfaioli B., Savarese I., Corciova S.A., Guerriero G., Lotti T. (2010). Cutaneous manifestations of breast carcinoma. Dermatol. Ther..

[15] Bastard D.P., Bollea-Garlatti M.L., Belatti A., Puga M.C., Hernández M.N., Mazzuoccolo L.D. (2019). Metástasis cutáneas de cáncer de mama: 8 años de revisión en un centro de tercera complejidad. Actas Dermosifiliogr..

[16] Ferlito A., Shaha A.R., Silver C.E., Rinaldo A., Mondin V. (2001). Incidence and Sites of Distant Metastases from Head and Neck Cancer. ORL.

[17] Nagi C., Bleiweiss I., Jaffer S. (2005). Epithelial displacement in breast lesions: A papillary phenomenon. Arch. Pathol. Lab. Med..

[18] Kovács K.A., Hegedus B., Kenessey I., Tímár J. (2013). Tumor type-specific and skin region-selective metastasis of human cancers: Another example of the “seed and soil” hypothesis. Cancer Metastasis Rev..

[19] Kang S.H., Kim W.S., Kim H.K., Bae T.H. (2022). Scalp metastasis from an adenocarcinoma of the lung that mimicked a cystic mass. Arch. Craniofac Surg..

[20] Coslett L.M., Katlic M.R. (1990). Lung cancer with skin metastasis. Chest.

[21] Mollet T.W., Garcia C.A., Koester G. (2009). Skin metastases from lung cancer. Dermatol. Online J..

[22] Alcaraz I., Cerroni L., Rütten A., Kutzner H., Requena L. (2012). Cutaneous metastases from internal malignancies: A clinicopathologic and immunohistochemical review. Am. J. Dermatopathol..

[23] Inamura K. (2018). Update on immunohistochemistry for the diagnosis of lung cancer. Cancers.

[24] Ward R.E., Ali S.A., Kuhar M. (2017). Epithelioid malignant mesothelioma metastatic to the skin: A case report and review of the literature. J. Cutan. Pathol..

[25] Mori T., Yamamoto T. (2021). Skin metastasis of malignant mesothelioma. An. Bras. Dermatol..

[26] Elbahaie A.M., Kamel D.E., Lawrence J., Davidson N.G. (2009). Late cutaneous metastases to the face from malignant pleural mesothelioma: A case report and review of the literature. World J. Surg. Oncol..

[27] Müller C.S.L., Reichrath J., Tilgen W. (2009). Disseminated cutaneous metastasis of a biphasic pleural mesothelioma. J. Eur. Acad. Dermatol. Venereol..

[28] Maiorana A., Giusti F., Cesinaro A.M., Conti A., Rossi G. (2006). Cutaneous metastases as the first manifestation of pleural malignant mesothelioma. J. Am. Acad. Dermatol..

[29] Beer T.W., Heenan P.J. (2007). Malignant mesothelioma presenting as a lip tumor: Report of two cases with one unrecognized by 166 pathologists. Am. J. Dermatopathol..

[30] Boyde A.M., Attanoos R.L. (2003). Sister Mary Joseph’s nodule in malignant peritoneal mesothelioma. Histopathology.

[31] Heatley M.K. (2004). Sister Mary Joseph’s nodule in malignant mesothelioma. Histopathology.

[32] Kanbay A., Oguzulgen K.I., Ozturk C., Memis L., Demircan S., Kurkcuoglu C., Akyurek N., Kurul C. (2007). Malignant pleural mesothelioma with scalp, cerebellar, and finger metastases: A rare case. South. Med. J..

[33] Cassarino D.S., Xue W., Shannon K.J. (2003). Widespread cutaneous and perioral metastases of mesothelioma. J. Cutan. Pathol..

[34] Dutt P.L., Baxter J.W., O’Malley F.P., Glick A.D., Page D.L. (1992). Distant cutaneous metastasis of pleural malignant mesothelioma. J. Cutan. Pathol..

[35] Shieh S., Grassi M., Schwarz J.K., Cheney R.T. (2004). Pleural mesothelioma with cutaneous extension to chest wall scars. J. Cutan. Pathol..

[36] Saeed S., Keehn C.A., Morgan M.B. (2004). Cutaneous metastasis: A clinical, pathological, and immunohistochemical appraisal. J. Cutan. Pathol..

[37] Nesseris I., Tsamakis C., Gregoriou S., Ditsos I., Christofidou E., Rigopoulos D. (2013). Cutaneous metastasis of colon adenocarcinoma: Case report and review of the literature. An. Bras. Dermatol..

[38] AlSubait N.A., BinJadeed H.F., AlSaleh M.R., AlFaifi F.S., AlSaif F.M., Arafah M.A. (2021). Dermoscopy of scalp cutaneous metastasis of sigmoid adenocarcinoma. JAAD Case Rep..

[39] Stephens K.R., Donica W.R.F., Egger M.E., Philips P., Scoggins C.R., McMasters K.M., Martin R.C.G. (2025). Observed changes in the distribution of colon cancer metastasis: A National Cancer Database review institutional experience. Ann. Surg. Oncol..

[40] Wong C.Y., Helm M.A., Helm T.N., Zeitouni N. (2014). Patterns of skin metastases: A review of 25 years’ experience at a single cancer center. Int. J. Dermatol..

[41] Şahin M., Ekinci F., Çelik C., Temiz P., Erdoğan A.P., Göksel G. (2021). A rare case report of skin metastasis in gastric cancer. J. Gastrointest. Cancer.

[42] Frey L., Vetter-Kauczok C., Gesierich A., Bröcker E.B., Ugurel S. (2009). Cutaneous metastases as the first clinical sign of metastatic gastric carcinoma. J. Dtsch. Dermatol. Ges..

[43] Takata T., Takahashi A., Tarutani M., Sano S. (2014). A rare case of cellulitis-like cutaneous metastasis of gastric adenocarcinoma. Int. J. Dermatol..

[44] Koo D.H., Chang H.M., Jung J.Y., Song J.H., Lee J.L., Ryu M.H., Kim T.W., Yook J.H., Song J.S., Lee J.S. (2007). Cutaneous metastasis resembling acute dermatitis in patient with advanced gastric cancer. Clin. Exp. Dermatol..

[45] Hu S.C., Chen G.S., Wu C.S., Chai C.Y., Chen W.T., Lan C.C. (2009). Rates of cutaneous metastases from different internal malignancies: Experience from a Taiwanese medical center. J. Am. Acad. Dermatol..

[46] Pliakou E., Lampropoulou D.I., Nasi D., Aravantinos G. (2022). Skin metastases from gastric cancer, a rare entity masquerading as erysipelas: A case report. Mol. Clin. Oncol..

[47] Zhou H.Y., Wang X.B., Gao F., Bu B., Zhang S., Wang Z. (2014). Cutaneous metastasis from pancreatic cancer: A case report and systematic review of the literature. Oncol. Lett..

[48] Kaoutzanis C., Chang M.C., Abdul Khalek F.J., Kreske E. (2013). Non-umbilical cutaneous metastasis of a pancreatic adenocarcinoma. BMJ Case Rep..

[49] Aghighi M., Bagher Shokravi M., Rahvar M. (2022). Metastatic pancreatic adenocarcinoma to umbilical skin. Cureus.

[50] Vekariya P., Daneti D.B., Senthamizh Selvan K., Verma S.K., Hamide A., Mohan P. (2020). Sister Mary Joseph nodule as an initial presentation of pancreatic adenocarcinoma. ACG Case Rep. J..

[51] Hafez H.Z. (2008). Cutaneous pancreatic metastasis: A case report and review of literature. Indian J. Dermatol..

[52] Miyahara M., Hamanaka Y., Kawabata A., Sato Y., Tanaka A., Yamamoto A., Ueno T., Nishihara K., Suzuki T. (1996). Cutaneous metastases from pancreatic cancer. Int. J. Pancreatol..

[53] Horino K., Takamori H., Ikuta Y., Nakahara O., Chikamoto A., Ishiko T., Beppu T., Baba H. (2012). Cutaneous metastases secondary to pancreatic cancer. World J. Gastrointest. Oncol..

[54] Gu L., Mehta P.P., Rao D., Rotemberg V., Capanu M., Chou J., Lin S., Sigel C.S., Busam K.J., Boyce L. (2023). Pancreatic cancer: Cutaneous metastases, clinical descriptors and outcomes. Cancer Med..

[55] Reuben S., Owen D., Lee P., Weiss A. (2009). Hepatocellular carcinoma with cutaneous metastases. Can. J. Gastroenterol..

[56] Peters R.L., Okuda K., Peters R.L. (1976). Metastatic patterns of HCC. Hepatocellular Carcinoma.

[57] Cazzato G., Colagrande A., Cimmino A., De Marco A., Romita P., Foti C., Resta L., Ingravallo G. (2021). Cutaneous metastases from primary liver cancers: The need for knowledge and differential diagnosis. Life.

[58] Morishita A., Tani J., Oura K., Tadokoro T., Fujita K., Masaki T. (2022). Giant cutaneous metastasis from hepatocellular carcinoma. JGH Open.

[59] Ackerman D., Barr R.J., Elias A.N. (2001). Cutaneous metastases from hepatocellular carcinoma. Int. J. Dermatol..

[60] Tümen D., Heumann P., Gülow K., Demirci C.N., Cosma L.S., Müller M., Kandulski A. (2022). Pathogenesis and Current Treatment Strategies of Hepatocellular Carcinoma. Biomedicines.

[61] Hennedige T., Venkatesh S.K. (2013). Imaging of hepatocellular carcinoma: Diagnosis, staging and treatment monitoring. Cancer Imaging.

[62] Amador A., Monforte N.G., Bejarano N., Martí J., Artigau E., Navarro S., Fuster J. (2007). Cutaneous metastasis from hepatocellular carcinoma as the first clinical sign. J. Hepatobiliary Pancreat. Surg..

[63] Hanazawa T., Fukami Y., Osawa T., Kurahashi S., Matsumura T., Saito T., Komatsu S., Kaneko K., Tsuzuki T., Sano T. (2021). A case of resected hepatocellular carcinoma with gallbladder metastasis. Surg. Case Rep..

[64] de Agustín P., Conde E., Alberti N., Pérez-Barrios A., López-Ríos F. (2007). Cutaneous metastasis of occult hepatocellular carcinoma: A case report. Acta Cytol..

[65] Wee A. (2011). Fine needle aspiration biopsy of hepatocellular carcinoma and hepatocellular nodular lesions: Role, controversies and approach to diagnosis. Cytopathology.

[66] Sheefa H., Lata J., Basharat M., Rumana M., Veena M. (2016). Utility of FNAC in conjunction with cell block for diagnosing space-occupying lesion (SOL) of liver with emphasis on differentiating hepatocellular carcinoma from metastatic SOL: Analysis of 61 Cases. Oman Med. J..

[67] Nadal R., Bellmunt J. (2019). Management of metastatic bladder cancer. Cancer Treat. Rev..

[68] Hasan O., Houlihan M., Wymer K., Hollowell C.M.P., Kohler T.S. (2019). Cutaneous metastasis of bladder urothelial carcinoma. Urol. Case Rep..

[69] Block C.A., Dahmoush L., Konety B.R. (2006). Cutaneous metastases from transitional cell carcinoma of the bladder. Urology.

[70] Baczyński A., Howard N., Tomaszewski J.J. (2023). Cutaneous metastasis of urothelial carcinoma. Dermatol. Pract. Concept..

[71] Vasilevska D., Rudaitis V., Lewkowicz D., Širvienė D., Mickys U., Semczuk M., Obrzut B., Semczuk A. (2024). Expression patterns of cytokeratins (CK7, CK20, CK19, CK AE1/AE3) in atypical endometrial hyperplasia coexisting with endometrial cancer. Int. J. Mol. Sci..

[72] Krathen R.A., Orengo I.F., Rosen T. (2003). Cutaneous metastasis: A meta-analysis of data. South. Med. J..

[73] Kirkali Z., Chan T., Manoharan M., Algaba F., Busch C., Cheng L., Kiemeney L., Kriegmair M., Montironi R., Murphy W.M. (2005). Bladder cancer: Epidemiology, staging and grading, and diagnosis. Urology.

[74] Manzelli A., Quaresima S., Rossi P., Petrou A., Ricciardi E., Brennan N., Kontos M., Petrella G. (2011). Solitary skin metastasis from sarcomatoid carcinoma of the bladder: A case report. J. Med. Case Rep..

[75] Mitsui Y., Arichi N., Inoue K., Hiraki M., Nakamura S., Hiraoka T., Ishikawa N., Maruyama R., Yasumoto H., Shiina H. (2014). Choroidal and cutaneous metastasis from urothelial carcinoma of the bladder after radical cystectomy: A case report and literature review. Case Rep. Urol..

[76] Tonni E., Oltrecolli M., Pirola M., Tchawa C., Roccabruna S., D’Agostino E., Matranga R., Piombino C., Pipitone S., Baldessari C. (2024). New Advances in Metastatic Urothelial Cancer: A Narrative Review on Recent Developments and Future Perspectives. Int. J. Mol. Sci..

[77] Brown G.T., Patel V., Lee C.C. (2014). Cutaneous metastasis of prostate cancer: A case report and review of the literature with bioinformatics analysis of multiple healthcare delivery networks. J. Cutan. Pathol..

[78] Wang S.Q., Mecca P.S., Myskowski P.L., Slovin S.F. (2008). Scrotal and penile papules and plaques as the initial manifestation of a cutaneous metastasis of adenocarcinoma of the prostate: Case report and review of the literature. J. Cutan. Pathol..

[79] Rattanasirivilai A., Kurban A., Lenzy Y.M., Yaar R. (2011). Cutaneous metastasis of prostatic adenocarcinoma: A cautionary tale. J. Cutan. Pathol..

[80] Lookingbill D.P., Spangler N., Sexton F.M. (1990). Skin involvement as the presenting sign of internal carcinoma. A retrospective study of 7316 cancer patients. J. Am. Acad. Dermatol..

[81] Oh T.H., Kim H.S., Park S.C. (2023). Scalp nodules as the first presentation of prostate cancer: A CARE-compliant article. Medicine.

[82] Stanko C., Grandinetti L., Baldassano M., Mahmoodi M., Kantor G.R. (2007). Epidermotropic metastatic prostate carcinoma presenting as an umbilical nodule-Sister Mary Joseph nodule. Am. J. Dermatopathol..

[83] Gurel B., Ali T.Z., Montgomery E.A., Begum S., Hicks J., Goggins M., Eberhart C.G., Clark D.P., Bieberich C.J., Epstein J.I. (2010). NKX3.1 as a marker of prostatic origin in metastatic tumors. Am. J. Surg. Pathol..

[84] De Bruyne P., Schatteman P., De Naeyer G., Carpentier P., Mottrie A. (2015). Port site metastasis in prostate cancer. Can. Urol. Assoc. J..

[85] Larrousse C., Brasseur P., Sukkarieh F. (2005). Métastase orificielle après prostatectomie radicale coelioscopique pour un adénocarcinome mucineux de la prostate [Port-site metastasis following laparoscopic radical prostatectomy for mucinous adenocarcinoma of the prostate]. J. Radiol..

[86] Sarangi S.S., Singh V., Bhirud D.P. (2023). Cutaneous metastasis of castration-resistant prostate cancer: A rare case report and review of literature. Urol. Ann..

[87] Petcu E.B., Gonzalez-Serva A., Wright R.G., Slevin M., Brinzaniuc K. (2012). Prostate carcinoma metastatic to the skin as an extramammary Paget’s disease. Diagn. Pathol..

[88] Bailey C., Broadbent A. (2007). Cutaneous metastases of prostate cancer. J. Palliat. Med..

[89] Villalba Bachur Roberto F., Florencia C., Emilio L. (2020). Prostate cancer cutaneous metastasis: A case report. Urol. Androl. Open J..

[90] Mak G., Chin M., Nahar N., De Souza P. (2014). Cutaneous metastasis of prostate carcinoma treated with radiotherapy: A case presentation. BMC Res. Notes.

[91] Reingold I.M. (1966). Cutaneous metastases from internal carcinoma. Cancer.

[92] Cormio G., Capotorto M., Di Vagno G., Cazzolla A., Carriero C., Selvaggi L. (2003). Skin metastases in ovarian carcinoma: A report of nine cases and a review of the literature. Gynecol. Oncol..

[93] Tavares V., Marques I.S., Melo I.G., Assis J., Pereira D., Medeiros R. (2024). Paradigm Shift: A Comprehensive Review of Ovarian Cancer Management in an Era of Advancements. Int. J. Mol. Sci..

[94] Zhang J., He W., Zhang Z., Dong H., Deng X., Wen Q., Li D. (2024). Skin metastasis from ovarian cancer with somatic BRCA1 mutation: A case report and literature review. Oncol. Lett..

[95] Bayraktar E., Chen S., Corvigno S., Liu J., Sood A.K. (2024). Ovarian cancer metastasis: Looking beyond the surface. Cancer Cell..

[96] Singh R.P., Tullis S., Hatton M., Rubin P.A. (2006). Orbital metastasis from ovarian carcinoma in a patient with BRCA-2 mutation. Ophthalmic Plast. Reconstr. Surg..

[97] Patsner B., Mann W.J., Chumas J., Loesch M. (1988). Herpetiform cutaneous metastases following negative second look laparatomy for ovarian adenocarcinoma. Arch. Gynecol. Obstet..

[98] McDonald H.H., Moore M.R., Meffert J.J. (2016). Cutaneous metastases from adenocarcinoma of the ovary. JAAD Case Rep..

[99] Scheinfeld N. (2008). A review of the cutaneous paraneoplastic associations and metastatic presentations of ovarian carcinoma. Clin. Exp. Dermatol..

[100] Cheng B., Lu W., Wan X., Chen Y., Xie X. (2009). Extra-abdominal metastases from epithelial ovarian carcinoma: An analysis of 20 cases. Int. J. Gynecol. Cancer.

[101] Otsuka I., Matsuura T. (2017). Skin metastases in epithelial ovarian and fallopian tube carcinoma. Medicine.

[102] Otsuka I. (2019). Cutaneous metastases in ovarian cancer. Cancers.

[103] Cheng H., Gao C., Zhang R., Yang Z., Zhang G. (2017). Two independent incidences of skin metastases in the umbilicus and abdominal wall in ovarian serous adenocarcinoma: A case report and review of the literature. Medicine.

[104] Fujiwara M., Taube J., Sharma M., McCalmont T.H., Kim J. (2010). PAX8 discriminates ovarian metastases from adnexal tumors and other cutaneous metastases. J. Cutan. Pathol..

[105] Lalich D., Tawfik O., Chapman J., Fraga G. (2010). Cutaneous metastasis of ovarian carcinoma with shadow cells mimicking a primary pilomatrical neoplasm. Am. J. Dermatopathol..

[106] Charalampidis C., Lampaki S., Zarogoulidis P., Lazaridis G., Mpaka S., Kosmidis C., Tsakiridis K., Kioumis I., Pavlidis P., Karapantzos I. (2016). Fine-needle aspiration of skin metastasis in ovarian cancer-report of two cases and review of the literature. Ann. Transl. Med..

[107] Markowska A., Baranowski W., Pityński K., Chudecka-Głaz A., Markowska J., Sawicki W. (2023). Metastases and recurrence risk factors in endometrial cancer-the role of selected molecular changes, hormonal factors, diagnostic methods and surgery procedures. Cancers.

[108] Hussein M.R. (2010). Skin metastasis: A pathologist’s perspective. J. Cutan. Pathol..

[109] Debois J.M. (1982). Endometrial adenocarcinoma metastatic to the scalp. Report of two cases. Arch. Dermatol..

[110] Bashyam A., Stewart A., Potter K., Bagwan I., Sunkaraneni V.S. (2018). Metastatic endometrial cancer of the paranasal sinuses. Ann. R. Coll. Surg. Engl..

[111] Giardina V.N., Morton B.F., Potter G.K., Mesa-Tejada R., Waterfield W.C. (1996). Metastatic endometrial adenocarcinoma to the skin of a toe. Am. J. Dermatopathol..

[112] Espinós J.J., Garcia-Patos V., Guiu X.M., Delgado E. (1993). Early skin metastasis of endometrial adenocarcinoma: Case report and review of the literature. Cutis.

[113] Patel K.S., Watkins R.M. (1992). Recurrent endometrial adenocarcinoma presenting as an umbilical metastasis (Sister Mary Joseph’s nodule). Br. J. Clin. Pract..

[114] Galle P.C., Jobson V.W., Homesley H.D. (1981). Umbilical metastasis from gynecologic malignancies: A primary carcinoma of the fallopian tube. Obstet. Gynecol..

[115] Augustin G., Kekez T., Bogdanic B. (2010). Abdominal papular zosteriform cutaneous metastases from endometrial adenocarcinoma. Int. J. Gynaecol. Obstet..

[116] Meedimale P., Meedimale R.S., Narayen V., Chella V.R., Patil P., Bhende A., Panda J. (2023). An exceptional case of carcinoma cervix with distant metastasis to skin. J. Intern. Med. India.

[117] Agrawal A., Yau A., Magliocco A., Chu P. (2010). Cutaneous metastatic disease in cervical cancer: A case report. J. Obstet. Gynaecol. Can..

[118] Zhao X., Chen R., Zhou Q. (2023). Skin metastasis in squamous cell cancer of cervix: A case report and literature review. Precis. Radiat. Oncol..

[119] Alrefaie S.I., Alshamrani H.M., Abduljabbar M.H., Hariri J.O. (2019). Skin metastasis from squamous cell carcinoma of the cervix to the lower extremities: Case report and review of the literature. J. Fam. Med. Prim. Care.

[120] Dai Y., Zhang Y., Ke X., Liu Y., Zang C. (2023). Cutaneous metastasis from cervical cancer to the scalp and trunk: A case report and review of the literature. J. Med. Case Rep..

[121] Tharakaram S., Rajendran S.S., Premalatha S., Yesudian P., Zahara A. (1985). Cutaneous metastasis from carcinoma cervix. Int. J. Dermatol..

[122] Li H., Wu X., Cheng X. (2016). Advances in diagnosis and treatment of metastatic cervical cancer. J. Gynecol. Oncol..

[123] Daw E., Riley S. (1982). Umbilical metastasis from squamous carcinoma of the cervix: Case report. Br. J. Obstet. Gynaecol..

[124] Behtash N., Mehrdad N., Shamshirsaz A., Hashemi R., Amouzegar Hashemi F. (2008). Umblical metastasis in cervical cancer. Arch. Gynecol Obstet..

[125] Maheshwari G.K., Baboo H.A., Ashwathkumar R., Dave K.S., Wadhwa M.K. (2001). Scalp metastasis from squamous cell carcinoma of the cervix. Int. J. Gynecol Cancer..

[126] Takagi H., Miura S., Matsunami K., Ikeda T., Imai A. (2010). Cervical cancer metastasis to the scalp: Case report and literature review. Eur. J. Gynaecol. Oncol..

[127] Kovács K.A., Kenessey I., Tímár J. (2013). Skin metastasis of internal cancers: A single institution experience. Pathol. Oncol. Res..

[128] Hsieh J.J., Purdue M.P., Signoretti S., Swanton C., Albiges L., Schmidinger M., Heng D.Y., Larkin J., Ficarra V. (2017). Renal cell carcinoma. Nat. Rev. Dis. Primers..

[129] Errami M., Margulis V., Huerta S. (2016). Renal cell carcinoma metastatic to the scalp. Rare Tumors.

[130] Snow S., Madjar D., Reizner G., Mack E., Bentz M. (2001). Renal cell carcinoma metastatic to the scalp: Case report and review of the literature. Dermatol. Surg..

[131] Mueller T.J., Wu H., Greenberg R.E., Hudes G., Topham N., Lessin S.R., Uzzo R.G. (2004). Cutaneous metastases from geniourinary malignancies. Urology.

[132] Lee H.J., Lee A., Tan D., Du J., Wang Y., Tang P.Y., Sim A.S.P. (2020). Cutaneous metastasis of renal cell carcinoma. Lancet Oncol..

[133] Leve P.P., Felício J., Carneiro R., Zagalo C. (2021). Recurrent renal cell carcinoma presenting as a cutaneous metastasis: A case report and review of the literature. Urol. Ann..

[134] Bujons A., Pascual X., Martínez R., Rodríguez O., Palou J., Villavicencio H. (2008). Cutaneous metastases in renal cell carcinoma. Urol. Int..

[135] de Paula T.A., da Silva P.S., Berriel L.G. (2010). Renal cell carcinoma with cutaneous metastasis: Case report. J. Bras. Nefrol..

[136] Ferhatoglu M.F., Senol K., Filiz A.I. (2018). Skin metastasis of renal cell carcinoma: A case report. Cureus.

[137] Singh P., Somani K. (2020). Latent distant metastasis of renal cell carcinoma to skin: A case report. Clin. Case Rep..

[138] Kume H., Teramoto S., Kitamura T. (2009). Metachronous bilateral renal cell carcinoma with an interval of more than 10 years. Int. Urol. Nephrol..

[139] Pitman K.T., Johnson J.T. (1999). Skin metastases from head and neck squamous cell carcinoma: Incidence and impact. Head Neck.

[140] Kmucha S.T., Troxel J.M. (1993). Dermal metastases in epidermoid carcinoma of the head and neck. Arch. Otolaryngol. Head. Neck Surg..

[141] Johnson D.E., Burtness B., Leemans C.R., Lui V.W.Y., Bauman J.E., Grandis J.R. (2020). Head and neck squamous cell carcinoma. Nat. Rev. Dis. Primers.

[142] Crosetti E., Arrigoni G., Cerutti M., Succo G. (2020). Atypical carcinoid of the larynx. Ear Nose Throat J..

[143] Thariat J., Badoual C., Hans S., Meatchi T., Housset M. (2008). Skin metastasis of head and neck carcinoma predictive for dismal outcome. Dermatol. Online J..

[144] El Khoury J., Khalifeh I., Kibbi A.G., Abbas O. (2014). Cutaneous metastasis: Clinicopathological study of 72 patients from a tertiary care center in Lebanon. Int. J. Dermatol..

[145] Gor D., Ghimire B., Abbas O., Diab M. (2025). Cutaneous metastases without a known primary: A clinical conundrum. Cureus.

[146] Virmani N.C., Sharma Y.K., Panicker N.K., Dash K.N., Patvekar M.A., Deo K.S. (2011). Zosteriform skin metastases: Clue to an undiagnosed breast cancer. Indian. J. Dermatol..

[147] Savoia P., Fava P., Deboli T., Quaglino P., Bernengo M.G. (2009). Zosteriform cutaneous metastases: A literature meta-analysis and a clinical report of three melanoma cases. Dermatol. Surg..

[148] Dumlao J.K.G., Cubillan E.L.A., Villena J.P.D.S. (2022). Clinical and histopathologic profile of patients with cutaneous metastasis in a tertiary hospital in the Philippines. Dermatopathology.

[149] Bolognia J.L., Schaffer J.V., Cerroni L. (2018). Dermatology.

[150] de Carvalho M., Valejo Coelho M.M., Bajanca R. (2025). Metástases cutâneas de carcinoma de células renais: A propósito de dois casos clínicos. Acta Med. Port..

[151] Spratt D.E., Gordon Spratt E.A., Wu S., DeRosa A., Lee N.Y., Lacouture M.E., Barker C.A. (2014). Efficacy of skin-directed therapy for cutaneous metastases from advanced cancer: A meta-analysis. J. Clin. Oncol..

[152] Russano F., Del Fiore P., Di Prata C., Pasqual A., Marconato R., Campana L.G., Spina R., Gianesini C.M., Collodetto A., Tropea S. (2021). The role of electrochemotherapy in the cutaneous and subcutaneous metastases from breast cancer: Analysis of predictive factors to treatment from an Italian cohort of patients. Front. Oncol..

[153] Ágoston D., Baltás E., Ócsai H., Rátkai S., Lázár P.G., Korom I., Varga E., Németh I.B., Dósa-Rácz Viharosné É., Gehl J. (2020). Evaluation of calcium electroporation for the treatment of utaneous metastases: A double blinded randomised controlled phase II trial. Cancers.

[154] Morley J., Grocott P., Purssell E., Murrells T. (2019). Electrochemotherapy for the palliative management of cutaneous metastases: A systematic review and meta-analysis. Eur. J. Surg. Oncol..

[155] Hennequin C., Belkacémi Y., Bourgier C., Cowen D., Cutuli B., Fourquet A., Hannoun-Lévi J.M., Pasquier D., Racadot S., Rivera S. (2022). Radiotherapy of breast cancer. Cancer Radiother..

[156] Nakamura N., Kawamori J., Takahashi O., Shikama N., Sekiguchi K., Takahashi T., Kato S., Ogita M., Motegi A., Akimoto T. (2018). Palliative radiotherapy for breast cancer patients with skin invasion: A multi-institutional prospective observational study. Jpn. J. Clin. Oncol..

[157] Niu L., Mu F., Zhang C., Li Y., Liu W., Jiang F. (2013). Cryotherapy protocols for metastatic breast cancer after failure of radical surgery. Cryobiology.

[158] Yuan Y., Niu L., Mu F., Wang X., Zeng J., Yao F., Jiang F., He L., Chen J., Li J. (2013). Therapeutic outcomes of combining cryotherapy, chemotherapy and DC-CIK immunotherapy in the treatment of metastatic non-small cell lung cancer. Cryobiology.

[159] Xu J., Niu L., Mu F., Liu S., Leng Y., Liao M., Zeng J., Yao F., Chen J., Li J. (2013). Percutaneous comprehensive cryoablation for metastatic esophageal cancer after failure of radical surgery. Cryobiology.

[160] Chen F.Z., Zhao X.K. (2013). Prostate cancer: Current treatment and prevention strategies. Iran. Red. Crescent Med. J..

[161] Liao Y.-Y., Tsai C.-L., Huang H.-P. (2025). Optimizing Osimertinib for NSCLC: Targeting Resistance and Exploring Combination Therapeutics. Cancers.

[162] Ahn J., Nagasaka M. (2023). Spotlight on Cemiplimab-rwlc in the Treatment of Non-Small Cell Lung Cancer (NSCLC): Focus on Patient Selection and Considerations. Cancer Manag. Res..

[163] Swain S.M., Shastry M., Hamilton E. (2023). Targeting HER2-positive breast cancer: Advances and future directions. Nat. Rev. Drug Discov..

[164] Pavlovic D., Niciforovic D., Papic D., Milojevic K., Markovic M. (2023). CDK4/6 inhibitors: Basics, pros, and major cons in breast cancer treatment with specific regard to cardiotoxicity—A narrative review. Ther. Adv. Med. Oncol..

[165] Gion M., Blancas I., Cortez-Castedo P., Cortés-Salgado A., Marmé F., Blanch S., Morales S., Díaz N., Calvo-Plaza I., Recalde S. (2025). Atezolizumab plus paclitaxel and bevacizumab as first-line treatment of advanced triple-negative breast cancer: The ATRACTIB phase 2 trial. Nat. Med..

[166] Crawford E.D., Heidenreich A., Lawrentschuk N., Tombal B., Pompeo A.C.L., Mendoza-Valdes A., Miller K., Debruyne F.M.J., Klotz L. (2019). Androgen-targeted therapy in men with prostate cancer: Evolving practice and future considerations. Prostate Cancer Prostatic Dis..

[167] de Bono J., Mateo J., Fizazi K., Saad F., Shore N., Sandhu S., Chi K.N., Sartor O., Agarwal N., Olmos D. (2020). Olaparib for metastatic castration-resistant prostate cancer. N. Engl. J. Med..

[168] Sartor O., de Bono J., Chi K.N., Fizazi K., Herrmann K., Rahbar K., Tagawa S.T., Nordquist L.T., Vaishampayan N., El-Haddad G. (2021). VISION Investigators. Lutetium-177-PSMA-617 for Metastatic Castration-Resistant Prostate Cancer. N. Engl. J. Med..

[169] Doleschal B., Petzer A., Rumpold H. (2022). Current concepts of anti-EGFR targeting in metastatic colorectal cancer. Front. Oncol..

[170] Tabernero J., Grothey A., Van Cutsem E., Yaeger R., Wasan H., Yoshino T., Desai J., Ciardiello F., Loupakis F., Hong Y.S. (2021). Encorafenib Plus Cetuximab as a New Standard of Care for Previously Treated BRAF V600E-Mutant Metastatic Colorectal Cancer: Updated Survival Results and Subgroup Analyses from the BEACON Study. J. Clin. Oncol..

[171] Zhao P., Li L., Jiang X., Li Q. (2019). Mismatch repair deficiency/microsatellite instability-high as a predictor for anti-PD-1/PD-L1 immunotherapy efficacy. J. Hematol. Oncol..

[172] Coutzac C., Funk-Debleds P., Cattey-Javouhey A., Desseigne F., Guibert P., Marolleau P., Rochefort P., de la Fouchardière C. (2023). Targeting HER2 in metastatic gastroesophageal adenocarcinomas: What is new?. Bull. Cancer.

[173] Liu J., Matulonis U.A. (2025). Update on PARP inhibitors for the treatment of ovarian cancer. Clin. Adv. Hematol. Oncol..

[174] Lorusso D., Colombo N., Dubot C., Cáceres M.V., Hasegawa K., Shapira-Frommer R., Salman P., Yañez E., Gümüş M., Olivera M. (2025). Pembrolizumab plus chemotherapy for advanced and recurrent cervical cancer: Final analysis according to bevacizumab use in the randomized KEYNOTE-826 study. Ann. Oncol..

[175] Liolis E., Mulita F., Koutras A., Makatsoris T., Sivolapenko G. (2024). Exploring Bevacizumab’s Role in Gynecological Cancers: An Up-to-Date Narrative Review Focusing on Ovarian Cancer. Mater. Sociomed..

[176] Krishnamurthy S., Ahmed I., Bhise R., Mohanti B.K., Sharma A., Rieckmann T., Paterson C., Bonomo P. (2022). The dogma of Cetuximab and Radiotherapy in head and neck cancer—A dawn to dusk journey. Clin. Transl. Radiat. Oncol..

[177] Ferris R.L., Blumenschein G., Fayette J., Guigay J., Colevas A.D., Licitra L., Harrington K., Kasper S., Vokes E.E., Even C. (2016). Nivolumab for Recurrent Squamous-Cell Carcinoma of the Head and Neck. N. Engl. J. Med..

[178] Hao W., Chang R., Liu J., Wang Y., Ren M., Xin K., Liu B., Xie J., Yang Y. (2024). Case report: A case of advanced gastric cancer with multiple skin metastases, with significant relief from immunotherapy. Front. Immunol..

[179] Li J.J., Xu P.F., Nie Y.L. (2025). Partial response to posterior line immunotherapy for more than 15 months in a pMMR patient with cutaneous metastasis of rectal carcinoma: A case report. Ther. Adv. Gastroenterol..

[180] Slominski R.M., Raman C., Chen J.Y., Slominski A.T. (2023). How cancer hijacks the body’s homeostasis through the neuroendocrine system. Trends Neurosci..

[181] Pandey S., Bradley L., Del Fabbro E. (2024). Updates in Cancer Cachexia: Clinical Management and Pharmacologic Interventions. Cancers.

